# Impacts from Land Use Pattern on Spatial Distribution of Cultivated Soil Heavy Metal Pollution in Typical Rural-Urban Fringe of Northeast China

**DOI:** 10.3390/ijerph14030336

**Published:** 2017-03-22

**Authors:** Wenbo Li, Dongyan Wang, Qing Wang, Shuhan Liu, Yuanli Zhu, Wenjun Wu

**Affiliations:** College of Earth Sciences, Jilin University, Changchun 130061, China; finehighman@sina.cn (W.L.); wangqing1106@sina.com (Q.W.); liush1211@163.com (S.L.); zhuyl16@mails.jlu.edu.cn (Y.Z.); wjwu16@mails.jlu.edu.cn (W.W.)

**Keywords:** heavy metal pollution, spatial regression, cultivated land, black soil region

## Abstract

Under rapid urban sprawl in Northeast China, land conversions are not only encroaching on the quantity of cultivated lands, but also posing a great threat to black soil conservation and food security. This study’s aim is to explore the spatial relationship between comprehensive cultivated soil heavy metal pollution and peri-urban land use patterns in the black soil region. We applied spatial lag regression to analyze the relationship between PLI (pollution load index) and influencing factors of land use by taking suburban cultivated land of Changchun Kuancheng District as an empirical case. The results indicate the following: (1) Similar spatial distribution characteristics are detected between Pb, Cu, and Zn, between Cr and Ni, and between Hg and Cd. The Yitong River catchment in the central region, and the residential community of Lanjia County in the west, are the main hotspots for eight heavy metals and PLI. Beihu Wetland Park, with a larger-area distribution of ecological land in the southeast, has low level for both heavy metal concentrations and PLI values. Spatial distribution characteristics of cultivated heavy metals are related to types of surrounding land use and industry; (2) Spatial lag regression has a better fit for PLI than the ordinary least squares regression. The regression results indicate the inverse relationship between heavy metal pollution degree and distance from long-standing residential land and surface water. Following rapid urban land expansion and a longer accumulation period, residential land sprawl is going to threaten cultivated land with heavy metal pollution in the suburban black soil region, and cultivated land irrigated with urban river water in the suburbs will have a higher tendency for heavy metal pollution.

## 1. Introduction

Suburbs are the conjunction between urban and rural settlements and are characterized by integration of agricultural production in miniature, disordered industrial manufacturing, and flowing residential settlements [1,2]. As the urban boundary rapidly sprawls, land conversions, along with the change in production, are not only encroaching on the quantity of cultivated lands, but also posing a threat to soil quality and food security [3,4]. Anthropogenic activities carried by different land use types are reported to release varying volumes of heavy metals [5,6,7]. Therefore, spatial variations in land use pattern will constitute one of the major artificial influences on the spatial redistribution of soil heavy metals. In addition, heavy metal pollution of cultivated land surrounded by different land use types is more likely to be gravely disturbed by local land use [8,9], particularly for cultivated land in the suburbs. Compared with the removal of heavy metal pollution, soil heavy metal prevention is always preferred. Only on the basis of understanding spatial relationships between land use patterns and soil heavy metal accumulation of cultivated land can we generate strategies and practices to alleviate negative effects caused by land use.

Traditional studies on heavy metal pollution of cultivated soils combined with land use includes investigating and evaluating heavy metal concentrations, analyzing potential sources by principal component analysis, and isotopic tracing of heavy metal outputs from potential sources [10,11,12,13,14]. However, location accuracy from potential sources or preliminary analysis of the relationship between soil heavy metal pollution and land use remain insufficient for reflecting the general spatial pollution pattern under the impacts of land use and supporting regional land management policy for soil protection. Kriging interpolation and spatial regression models are the proper methods for analyzing spatial patterns of heavy metal pollution and relationships between spatial data given the spatial association of research data are not neglected because soil heavy metal concentrations are related to their neighbors rather than being independent of each other [15,16]. Kriging is an interpolation method that gives the best linear unbiased prediction of the intermediate values and is broadly applied in the field of soil heavy metal pollution [6,17]. Spatial error models and geographically-weighted regressions have been performed to analyze the relationship between heavy metal concentrations and possible influencing factors, and they are considered to be more powerful tools for exploring spatial heterogeneity and have a better goodness-of-fit than conventional linear regression models [9,16]. However, apart from the lack of consideration for the soil background values, the results from single heavy metal regression with land use information are used for source apportionment and pollution management rather than evaluating pollutant release based on differing land use types [9,16,18]. Heavy metal pollution is triggered by wastewater irrigation, applying fertilizer, household refuse disposal, and other practices carried out on or around the cultivated land. Therefore, it is generally a combined pollution [19] and a comprehensive indicator representing the average releasing level of heavy metals is more practical for study.

Black soil is named according to the standard classification and codes for Chinese soil and is analogous to Mollisol. The black soil region of Northeast China is one of few black soil resources in the world that provides high soil fertility and massive grain production [20]. However, rapid urban sprawl in Northeast China is seriously challenging environmental conditions [21,22]. With the aim of analyzing the spatial relationship between comprehensive cultivated soil pollution and peri-urban land use patterns in the black soil region, a suburb of Kuancheng District in Changchun city (one of the major metropolis in the northeast black soil region) was taken as an empirical case. We performed the spatial regression for PLI (pollution load index) of heavy metals with influencing land use factors. The research results serve as a theoretical basis for land management policy that prevents heavy metal pollution in cultivated soil in the black soil region.

## 2. Materials and Methods

### 2.1. Study Area

**Figure 1:**
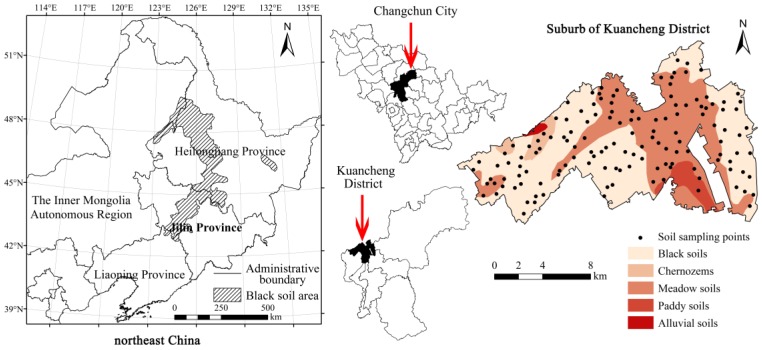
Distribution of the black soil region in Northeast China and a map of soil sampling points and soil types in a suburb of the Kuancheng District.

The study was carried out in a suburb of Changchun Kuancheng District (Figure 1), one of the typical rural-urban interfaces located in the black soil region of northeast China. The total area of the Kuancheng District is 23,800 ha, and the population is approximately 0.6 million. Kuancheng District is an old industrial area in Changchun City with the Yitong River flowing through; many boiler factories, electrical machinery plants, and pharmaceutical factories have been located in this district since the early 1950s. In addition, it belongs to the Jilin golden corn production belt, and there is a cluster of superior agricultural resources in this area. The contrast between industrial development and agricultural production is exceptional in this area. Moreover, land use in the study area changed significantly in recent years (see Supplementary Materials Table S1). The distribution of the narrow black soil region was mapped from an earlier paper [23], and the distribution of soil types in the study area was obtained from the results of the first soil general survey in China.

### 2.2. Soil Sampling and Chemical Analysis

A total of 137 soil samples (Figure 1) were collected from cultivated lands in a suburb of Kuancheng District. Sampling points were randomly distributed throughout the study area. Topsoil (0–20 cm) samples were collected with a stainless steel shovel and placed in cloth bags after litter removal. Each soil sample was composed of 3–5 sub-samples collected within 100 m of the corresponding sampling point, and all the sampling coordinates were recorded by portable GPS. Soil sampling was conducted in September 2015.

Soil samples were air-dried in the shade, ground, passed through a 2-mm plastic sieve and stored in sealed plastic bags prior to analysis. Total concentrations of Pb, Zn, Cu, Cr, and Ni were analyzed via X-ray fluorescence (XRF) after soil samples were pressed into pellets. The total concentration of Cd was analyzed via inductively-coupled plasma mass spectrometry (ICP-MS) method after a four-acid digestion (HNO_3_, HCl, HF, and HClO_4_). Total concentrations of As and Hg were analyzed via the atomic fluorescence spectrometry (AFS) method; soil was digested with chloroazotic acid and reduced by KBH_4_ solution prior to analysis. Thirteen certified reference material samples were processed and measured to assess analysis quality. Soil chemical analysis methods were conducted according to the specifications for national multi-purpose regional geochemical survey (NMPRGS) by the China Geological Survey, and analysis results are consistent with the analytical requirements developed in the NMPRGS (see Supplementary Materials Tables S2 and S3).

### 2.3. Statistical Analysis and Background Values

Descriptive statistics of soil heavy metal concentrations are presented in Table 1, background values for As, Hg, Cd, Pb, Cr, Ni, Cu, and Zn of different soil types are provided by the Study on Background Values of Soil Elements in Jilin Province [24]. As presented in Table 1, arithmetic mean values of heavy metal concentration exceed the background for the most part, which indicates potential for soil heavy metal accumulation. The coefficients of variation of Hg and Cd are 51.91% and 38.69%, respectively, and they are of greater spatial variability than the other elements.

### 2.4. Soil Pollution Assessment and Interpolation Methods

The PLI (pollution load index) for As, Hg, Cd, Pb, Cr, Ni, Cu, and Zn were applied to determine the pollution status of cultivated soil:

(1) $$C_{f}^{i}=C_{i}/C_{b}^{i},\;\mathrm{PLI}=\sqrt[{n}]{C_{f}^{1}\times C_{f}^{2}\times C_{f}^{3}\times \cdots \times C_{f}^{n}}$$

In Equation (1), where $C_{f}^{i}$ is the concentration factor; $C_{i}$ is the concentration of heavy metal; $C_{b}^{i}$ is the element background value; and PLI is the pollution load index. To eliminate the effects of soil type on heavy metal concentration, the background value (Table 1) applied is in accord with the soil type for each sample. PLI is the combined characterization for soil heavy metal combined pollution. It is a concise way to evaluate the status of soil heavy metal pollution [25].

To map the spatial distribution of soil heavy metal concentration and PLI, an ordinary Kriging interpolation was applied in this paper. Kriging interpolation is one of the best linear unbiased estimator methods for mapping soil properties [6]. It is adopted on the basis of the normal distribution test and semi-variogram fitting for regionalized variables. If the original data or the logarithmic transformed data had a normal distribution, ordinary Kriging was applied for interpolation maps. Otherwise, inverse distance weighted interpolation was applied. The spatial distribution maps of heavy metal concentrations and PLI were plotted using the ArcGIS (10.1, Environmental Systems Research Institute Inc., Redlands, CA, USA) Geostatistical Analyst module.

### 2.5. Information Extracted for Land Use and Spatial Regression Model

#### 2.5.1. Land Use Information Extracted

**Figure 2:**
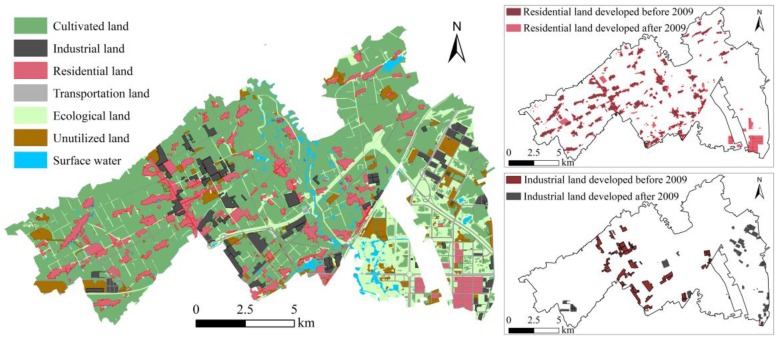
Spatial distribution map of land use types in the study area.

The spatial distribution of land use types in the study area was interpreted from high-resolution remote sensing images (IKONOS) in 2014 using ArcGIS (10.1, Environmental Systems Research Institute Inc.) along with a field survey. Land uses were classified into seven types: cultivated land (dry land, paddy land, and agricultural greenhouses), industrial land (land for warehousing, industrial manufacture and mining), residential land (urban and rural settlements), transportation land (highway and railway), ecological land (garden plot, grassland, and forest land for city greening or wind sheltering), unutilized land (bare land surface without covering of vegetation or constructions), and surface water (river and irrigation reservoirs). In consideration of the accumulative characteristics of soil heavy metals, two potential pollution sources and unstable land use types, industrial land and residential land, were both divided into two groups: land developed before 2009 and land developed after 2009 (Figure 2).

With the aim of gathering land use information for ecological land and providing spatial analysis, Thiessen polygons were constructed according to locations of sampling points. Spatial autocorrelation analysis and spatial auto-regression were performed on the basis of Thiessen polygons.

#### 2.5.2. Spatial Regression Model

Soil heavy metal pollution degree has certain spatial dependencies, including spatial autocorrelation. Therefore, simulation deviations will be created owing to spatial data autocorrelation for traditional regression analysis. To simulate the relationship between PLI and land use influencing factors preferentially, a spatial auto-regression model was adopted (Equation (2)). Among the four variant forms of spatial auto-regression models, spatial lag model and spatial error model are the most widely used, and they are the models discussed here in this paper:

(2) $$\begin{array}{l} y=\rho \omega_{1}y+x\beta +\mu \\ \mu =\lambda \omega_{2}\mu +\varepsilon \\ \varepsilon \sim N\left(0,\sigma^{2}I\right) \end{array}$$

In Equation (2), y is the dependent variable; x is the explanatory variable; ρ is the coefficient of spatial lag variable, $\omega_{1}y$; β is the parameter vector related to x; μ is the stochastic error term vector; λ is the coefficient of spatial error variable, $\omega_{2}\mu$; I is the unit matrix; and $\omega_{1}$ and $\omega_{2}$ are the weight matrices reflecting the spatial trend of the dependent variable and residual, respectively.

The spatial lag model shows that the dependent variable is under the influence of explanatory variables, locally, along with dependent variables in the neighborhood. The spatial error model shows that the dependent variable is under the common influence of local explanatory variables, dependent variables, and explanatory variables in the neighborhood [15,16].

Spatial model selection depends on the significance test results of the Lagrange multiplier for ordinary least squares (OLS) regression. There are four indicators of Lagrange multiplier statistics, Lagrange multiplier (lag), robust Lagrange multiplier (lag), Lagrange multiplier (error), and robust Lagrange multiplier (error). If the Lagrange multiplier (lag) and Lagrange multiplier (error) are both non-significant (*p*-value > 0.05), the results of OLS regression are retained. If only one of them is significant (*p*-value < 0.05), the model applied will correspond to the significant Lagrange multiplier, lag or error. If they are both significant, the one with lower *p*-value between the robust Lagrange multiplier (lag) and robust Lagrange multiplier (error) will determine the application of the spatial lag model or spatial error model [15]. Variables for spatial regression model are listed in Table 2.

## 3. Results

### 3.1. Spatial Structure and Distribution of Heavy Metals

As, Hg, Pb, Cr, Ni, and PLI data are all normally distributed, Cu data are normally distributed after a logarithmic transformation, and the remaining heavy metals do not conform to a normal distribution (see Supplementary Materials Table S4). However, the semi-variogram fitting for Pb was unsatisfactory; thus, inverse distance-weighted (IDW) interpolation was applied for Cd, Pb, and Zn. Concentrations of As, Hg, and logarithmic transformed Cu were fitted with an exponential model and Cr, Ni, and PLI were fitted with a spherical model (see Supplementary Materials Table S5 and Figure S1). R^2^ and residual sum of squares (RSS) indicate the logical fitting for heavy metals and the fitted semi-variogram models reflect their spatial structures (Table 3).

As shown in Table 3, C_0_/(C + C_0_) for As, Cr, Ni, Cu are all below 25%, which indicates strong spatial correlation and structural factors are the dominant influence on spatial variation. C_0_/(C + C_0_) for Hg and PLI are between 25% and 75%, which indicates moderate spatial correlation and the influence on spatial variation by structural factors and random factors are similar.

**Figure 3:**
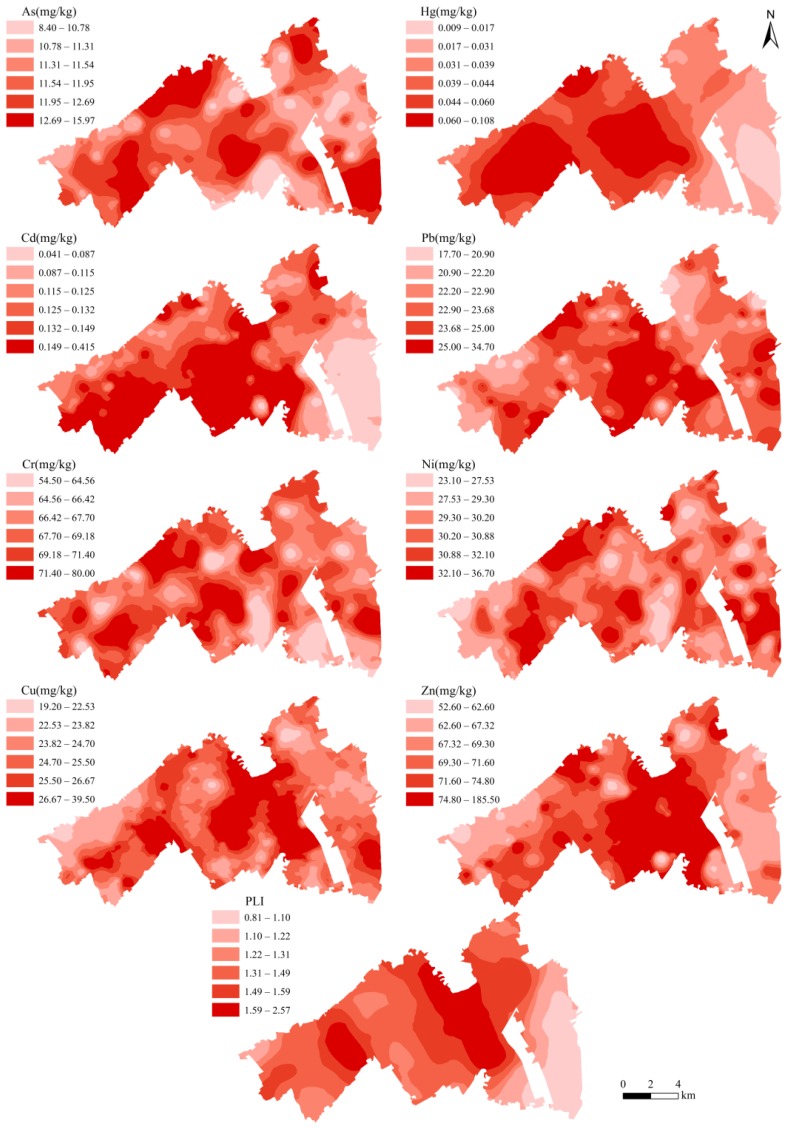
Spatial interpolation maps for heavy metals and PLI.

To increase the spatial differentiation for heavy metal concentrations, quantiles were used to classify each concentration range. Spatial interpolation maps for concentrations of As, Hg, Cd, Pb, Cr, Ni, Cu, and Zn are presented in Figure 3. As shown, similar spatial distribution characteristics could be detected between Pb, Cu, and Zn, between Cr and Ni, and between Hg and Cd. By comparing the heavy metal data to the map of land use types, we observe that the Yitong River catchment in the central region, and Lanjia County residential community in the west, are the main hotspots for eight heavy metals. Beihu Wetland Park, with a larger area distribution of ecological land in the southeast, has the lowest concentrations of heavy metals.

### 3.2. Effects of Land Use Pattern on Heavy Metal Pollution of Cultivated Land

To quantify the effects of land use pattern on heavy metal pollution of cultivated land, a spatial regression model was applied to analyze the relationship between heavy metal pollution and influencing land use factors.

Before spatial regression, OLS regression for PLI was performed for primary analysis and determination of spatial regression model type (Table 4). The OLS regression results indicate that RL_DB09 and SW_D are the only significant variables (*p*-value < 0.05), and coefficients show negative relationships. With the spatial autocorrelation analysis of all variables for spatial regression and residuals of OLS regression for PLI (Table 5), we determined a relatively distinct spatial autocorrelation for the variables, especially for the industrial land, residential land, ecological land, and surface water factors. Spatial autocorrelation of residuals by OLS regression for PLI indicates that spatial regression analysis needs to be conducted to eliminate the impacts.

To decide which spatial regression model should be applied, a Lagrange multiplier test was carried out for the OLS regression results (Table 6). As shown, the Lagrange multiplier (lag) and Lagrange multiplier (error) are both significant (*p*-value < 0.05). In addition, the robust Lagrange multiplier (lag) is of relatively higher significance than the robust Lagrange multiplier (error), thus, the spatial lag model should be applied for the PLI spatial regression.

With the higher R^2^ and log likelihood values (Table 7), we conclude that the spatial lag model regression is better fitted for PLI than the ordinary least squares regression. In addition, the univariate *Moran’s I* for residuals of spatial lag regression is −0.0072, which indicates the loss of spatial autocorrelation. Without the spatial autocorrelation impact of residuals, as calculated, the coefficients and *p*-values of explanatory variables from spatial lag regression vary from those of ordinary least squares regression. Under such assumptions of spatial lag regression, the local PLI is under the influence of influencing land use factors in local along with the PLI in the neighborhood.

According to the significance test (Table 7), among all explanatory variables, only RL_DB09 (distance from the sampling point to the nearest residential land developed before 2009) and SW_D (distance from the sampling point to the nearest river or irrigation reservoir) are significant (*p*-value < 0.05). This relationship indicates that long-standing residential land and agricultural irrigation pollution by surface water are the major influencing factors driving soil heavy metal pollution of suburban cultivated land. The regression coefficients (−4.08 × 10^−5^ and −9.30 × 10^−5^) represent the inverse relationship between heavy metal pollution degree and distance from long standing residential land and sources of surface irrigation water, respectively. To model a better fitting regression relationship, all non-significant redundant variables were removed; this model is hereafter known as spatial lag model B. All explanatory variables of spatial lag model B are significant, which indicates that it has a better fit for the regression than spatial lag model A.

## 4. Discussion

### 4.1. Spatial Distribution Characteristics of Heavy Metal Pollution in a Rural-Urban Fringe

Types and density of anthropogenic activities carried by different land use types are diverse; soil heavy metal concentrations vary with the land use type on account of the discrepant output of heavy metals [7,26]. Suburbs are the outer circles of urban expansion with dramatic land use change. Due to urban sprawl, a great area of cultivated land has been converted to urban construction land. Along a rural-urban gradient, distributions of industrial manufacturing, traffic networks, and population density are more scattered and more scarce from suburb to exurb [27,28]. Moreover, along with industry transformation and upgrading, an increasing number of secondary industry units emigrate from urban built-up areas and move to suburbs at the expense of cultivated land loss. Thus, not only the quantity of cultivated land has been reduced, but also the agricultural production activities have changed form, and human activities will continue to modify cultivated land [29]. Pollutant variation indicates different heavy metal output environments for cultivated soil, which will potentially prompt the distribution of cultivated land heavy metals into certain spatial variation patterns. It has been proved by previous studies that soil heavy metal concentration generally varies with the rural-urban gradient [30,31,32].

As shown in Figure 3, it can be observed that the Yitong River catchment in the central and Lanjia County residential community in the west are the main hotspots for eight heavy metals and PLI. A cluster of long-standing residential land and heavy industrial land in Lanjia County residential community appears to possess added volumes of pollutant release, and polluted water from the Yitong River is another determinant of cultivated soil pollution. However, the central area, where a large industrial area is located, has a lower value of PLI. The abnormity may be attributed to the fact that most industrial lands in this area are used for warehousing and agricultural product packaging, which have lower pollutant release than heavy industries. The construction project of Beihu Wetland Park started in 2010 has resulted in a surge of ecological land and local environmental improvement. It is prominent that Beihu Wetland Park, with a larger area distribution of ecological land in the southeast, has low levels for both heavy metal concentrations and PLI values. In addition, distribution characteristics of concentrations of As, Cr, Ni, Pb, Zn, and Cu in the southeast are different from Hg and Cd. Utilization and improper discarding of paint, cement, and other building materials for residential land developed in this area after 2009 may be the major anthropogenic sources of these elements [5]. However, low PLI value indicates that general accumulation of heavy metals in this area is not critical. To sum up, spatial distribution characteristics of cultivated heavy metals are related to types of surrounding land use and industry, and land use along a rural-urban gradient will prompt the distribution of cultivated land heavy metals into certain spatial variation patterns.

### 4.2. Impacts from Land Use Pattern on Cultivated Soil Heavy Metal Pollution

According to spatial regression results (Table 7), residential land developed before 2009 and surface water are the major influencing factors of land use increasing soil heavy metal pollution of suburban cultivated land. Compared to newly-developed residential land, long-standing rural settlement areas in Northeast China are short of resources and management for refuse disposal. Without the buffering effects of artificial ecological land, cultivated land surrounding rural settlements is more likely to be exposed to the direct pollution sources. Beyond that, according to our field survey, there is more potential for the cultivated lands near newly-collected cultivated land for development or future development to be incorporated shortly. That leads directly to the abandonment of cultivation by the landowner, and they are more inclined to outsource these cultivated lands at low rent to mitigate the risk of land loss and crop removal prior to harvest. In addition, these ‘cultivated lands at risk’ are generally absent tillage practices, such as applying compound fertilizer after seeding and frequent agricultural machinery practices, which will certainly block heavy metal inputs from agricultural sources. More importantly, heavy metal emission into long-standing residential land has a longer period for the soil accumulation. Newly-developed residential land, in contrast, is primarily converted from collected cultivated land. Thus, although newly-developed residential land is bearing a higher density population and traffic, in the short term, the PLI of cultivated land closer to long-standing residential land exceeds that of the newly-developed land. However, following rapid urban land expansion and a longer accumulation period, the residential land sprawl will threaten the cultivated land with heavy metal pollution in suburban black soil regions.

In addition, according to the spatial distribution characteristics of heavy metal pollution, we conclude that the heavy metal pollution of cultivated land in the Yitong River catchment and its branches are more severe than other areas. The Changchun section of the Yitong River has been reported on several times for poor water quality and potential heavy metal pollution [33]. There are many paddy lands along the riverbanks, which are primarily irrigated by water from the Yitong River or irrigation reservoirs directly replenished by the Yitong River. Since untreated domestic and industrial waste are discharged into water bodies, urban river systems are generally confronted with the risk of more heavy metal absorption [34,35]. In addition, many urban sections of rivers flowing through the black soil region of Northeast China have been reported as polluted by heavy metals [33,36,37,38]. Unlike exurban cultivated land, suburban cultivated land shares a greater proportion of urban river irrigation, which is more likely to be polluted by heavy metals due to shortages of irrigation and drainage facilities. As urbanization continues, heavy metals will drain into the river system in the black soil region and suburban cultivated land irrigated with urban river water will be a hot spot for heavy metal pollution.

## 5. Conclusions

This study analyzed the relationship between spatial distribution characteristics of heavy metal pollution and land use patterns of suburbs in a black soil region using spatial lag regression. The empirical case study area was cultivated land of suburban Kuancheng District, north of Changchun, China. Heavy metal pollution shows spatial characteristics in a rural-urban fringe. Similar spatial distribution characteristics are detected between Pb, Cu, and Zn, between Cr and Ni, and between Hg and Cd. In a comparison with the map of land use types, the Yitong River catchment in the central region and Lanjia County residential community in the west are the main hotspots for eight heavy metals and PLI. Beihu Wetland Park, with a larger area distribution of ecological land in the southeast, has the lowest concentrations of heavy metals. Spatial distribution characteristics of cultivated heavy metals are related to types of surrounding land use and industry. Moreover, spatial lag model regression provides better fitting for PLI than the ordinary least squares regression, and removes the spatial autocorrelation impact of residuals. Regression results indicate an inverse relationship between heavy metal pollution and the distance from long-standing residential land and surface water. Following rapid urban land expansion and longer accumulation periods, residential land sprawl is going to threaten cultivated land with heavy metal pollution in the suburban black soil region; suburban cultivated land irrigated with urban river water is more likely to be polluted by heavy metals.

## Figures and Tables

**Table 1:** Statistics of soil heavy metal concentrations and their background values (mg/kg) for different soil types.

Heavy Metals	Minimum	Maximum	Mean	CV %	Skewness	Kurtosis	Background Values for Heavy Metals in Jilin Province			
							Black Soil	Chernozem	Meadow Soil	Paddy Soil
As	8.40	15.97	11.65	9.97	0.389	1.650	11.08	9.30	9.71	7.13
Hg	0.009	0.108	0.041	51.91	0.946	1.165	0.035	0.027	0.029	0.040
Cd	0.041	0.415	0.126	38.69	3.114	16.872	0.083	0.091	0.075	0.082
Pb	17.70	34.70	23.16	10.76	1.216	3.631	22.14	20.20	17.88	23.60
Cr	54.50	80.00	67.87	5.98	0.108	1.040	52.94	30.86	41.31	50.10
Ni	23.10	36.70	29.96	8.36	−0.253	0.499	25.19	15.24	17.53	24.87
Cu	19.20	39.50	25.02	12.44	1.716	5.065	18.38	13.84	13.26	24.42
Zn	52.60	185.50	70.83	18.58	5.386	43.018	64.80	35.80	40.53	52.74

Note: CV, coefficient of variation. Soil types were named according to standard classification and codes for Chinese soil; Black soil belongs to the order of semi-luvisols; Chernozem belongs to the order of pedocal; Meadow soil belongs to the order of semi-hydromorphic soil; Paddy soil belongs to the order of anthrosols.

**Table 2:** Description of variables for the spatial regression model.

Variable Name	Variable Type	Units	Definition
PLI	dependent variable	-	Pollution load index, geometric mean value of heavy metal concentration factors
IL_DB09	explanatory variable	m	Distance from the sampling point to the nearest industrial land developed before 2009
IL_DA09	explanatory variable	m	Distance from the sampling point to the nearest industrial land developed after 2009
RL_DB09	explanatory variable	m	Distance from the sampling point to the nearest residential land developed before 2009
RL_DA09	explanatory variable	m	Distance from the sampling point to the nearest residential land developed after 2009
TL_D	explanatory variable	m	Distance from the sampling point to the nearest transportation land.
SW_D	explanatory variable	m	Distance from the sampling point to the nearest river or irrigation reservoir
EL_R	explanatory variable	%	Proportion of ecological land area to the total land area in every Thiessen polygon created according to the location of each sampling

**Table 3:** Parameters of semi-variogram fitting for heavy metals and validation results.

Element	Model	C_0_	C_0_ + C	Range	RSS	R^2^	C_0_/(C_0_ + C)
As	Exponential	0.227	1.441	2790	2.14 × 10^−1^	0.679	15.75%
Hg	Exponential	0.000239	0.000518	15,210	8.20 × 10^−9^	0.893	46.14%
Cd	Inverse distance weighted interpolation for concentration mapping						
Pb	Inverse distance weighted interpolation for concentration mapping						
Cr	Spherical	1.54	17.44	1640	20.2	0.706	8.83%
Ni	Spherical	0.16	6.231	1580	4.36	0.600	2.57%
Cu	Exponential	0.000404	0.002628	1380	9.88 × 10^−7^	0.176	15.37%
Zn	Inverse distance weighted interpolation for concentration mapping						
PLI	Spherical	0.025	0.0738	6150	5.10 × 10^−4^	0.843	33.88%

Note: RSS, residual sum of squares; C_0_, nugget variance; C + C_0_, sill variance; the ratio of C_0_/(C + C_0_) can reflect the spatial correlation degree of a regionalized variable: strong spatial correlation, C_0_/(C + C_0_) < 25%; moderate spatial correlation, 25% < C_0_/(C + C_0_) < 75%; and weak spatial correlation, C_0_/(C + C_0_) > 75%.

**Table 4:** Parameter statistics of ordinary least squares regression for PLI and significance test.

Regression Model	Variable	Coefficient	Std. Error	*t*-Value	*p*-Value
Ordinary least squares regression (R^2^: 0.3244 Log likelihood: 14.8794)	Constant	1.4961	0.0562	26.6017	0.0000
	IL_DB09	−2.18 × 10^−5^	1.17 × 10^−5^	−0.0850	0.0649
	IL_DA09	−1.59 × 10^−6^	1.87 × 10^−5^	0.4854	0.9324
	RL_DB09	−7.02 × 10^−5^	1.79 × 10^−5^	−3.9274	0.0001
	RL_DA09	1.70 × 10^−5^	1.48 × 10^−5^	1.1463	0.2538
	TL_D	−2.08 × 10^−5^	6.25 × 10^−5^	−0.3335	0.7393
	SW_D	−1.28 × 10^−4^	4.65 × 10^−5^	−2.7494	0.0068
	EL_R	−0.0003	0.0018	−0.1909	0.8489

**Table 5:** Univariate *Moran’s I* for influencing land use factors and residuals of OLS regression for PLI.

Variable	*n*	*Moran’s I*
PLI	137	0.4340
IL_DB09	137	0.8863
IL_DA09	137	0.7554
RL_DB09	137	0.9229
RL_DA09	137	0.8352
TL_D	137	0.4274
SW_D	137	0.5498
EL_R	137	0.7456
Residuals of OLS regression for PLI	137	0.1899

**Table 6:** Lagrange multiplier test statistics of OLS regression for PLI.

Lagrange Multiplier Test	*n*	*t*-Value	*p*-Value
Lagrange Multiplier (lag)	137	13.7457	0.0002
Robust Lagrange Multiplier (lag)	137	1.9309	0.1647
Lagrange Multiplier (error)	137	12.0476	0.0005
Robust Lagrange Multiplier (error)	137	0.2328	0.6295

**Table 7:** Parameter statistics of spatial lag model regression models for the PLI and significance test.

Regression Model	Variable	Coefficient	Std. Error	*z*-Value	*p*-Value
Spatial lag model A regression (R^2^: 0.4049; log likelihood: 21.0812)	W_PLI	0.4024	0.1032	3.9003	0.0001
	Constant	0.9128	0.1594	5.7276	0.0000
	IL_DB09	−1.25 × 10^−5^	1.09 × 10^−5^	−1.1414	0.2537
	IL_DA09	−1.85 × 10^−6^	1.71 × 10^−5^	−0.1086	0.9135
	RL_DB09	−4.08 × 10^−5^	1.76 × 10^−5^	−2.3128	0.0207
	RL_DA09	1.06 × 10^−5^	1.36 × 10^−5^	0.7822	0.4341
	TL_D	−3.52 × 10^−5^	5.69 × 10^−5^	−0.6175	0.5369
	SW_D	−9.30 × 10^−5^	4.28 × 10^−5^	−2.1716	0.0299
	EL_R	−0.0008	0.0016	−0.4882	0.6254
Spatial lag model B regression (R^2^: 0.3957; log likelihood: 19.7126)	W_PLI	0.4253	0.1013	4.2004	0.0000
	Constant	0.8532	0.1483	5.7538	0.0000
	RL_DB09	−4.66 × 10^−5^	1.42 × 10^−5^	−3.2670	0.0011
	SW_D	−8.66 × 10^−5^	4.14 × 10^−5^	−2.0945	0.0362

Note: W_PLI, spatial lag variable of PLI.

## Supplementary Materials

The following are available online at www.mdpi.com/1660-4601/14/3/336/s1, Figure S1: Semi-variograms for elements and PLI interpolation, Table S1: Land use area and percentage changes of study area from 2009 to 2014, Table S2: Statistics of chemical analysis for certified reference materials and qualification rate, Table S3: Statistics of chemical analysis for repeatedly-analyzed samples and qualification rate, Table S4: Statistics of Kolmogorov-Smirnov test for data of elements and PLI, Table S5: Statistics of semi-variogram fitting results for elements and PLI.
