# Supplementary material for: Impacts from Land Use Pattern on Spatial Distribution of Cultivated Soil Heavy Metal Pollution in Typical Rural-Urban Fringe of Northeast China
Source: Int J Environ Res Public Health. 2017 Mar 22;14(3):336. doi: 10.3390/ijerph14030336 (PMC5369171; doi:10.3390/ijerph14030336)
Supplement: Supplementary file 1 [file ijerph-14-00336-s001.pdf]

Supplementary Materials: Impacts from Land Use Patterns on the Spatial Distribution of Cultivated Soil Heavy Metal Pollution in Typical Rural-Urban Fringe of Northeast China

Wenbo Li, Dongyan Wang*, Qing Wang, Shuhan Liu, Yuanli Zhu, Wenjun Wu

Table S1. Land use area and percentage changes of study area from 2009 to 2014.

Land use type	Area (in ha)		Area changes (in ha)	Percentage changes (%)
	2009	2014	2009-2014	2009-2014
Cultivated land	13,792.88	11,042.08	–2750.8	–19.94
Residential land	1994.83	1990.03	–4.8	–0.24
Industrial land	529.07	1146	616.93	116.61
Transportation land	407.63	807.73	400.09	98.15
Ecological land	1286.71	2529.61	1242.9	96.6
Water	567.1	539.7	–27.4	–4.83
Unutilized land	482.38	1005.45	523.07	108.44

Table S2. Statistics of chemical analysis for certified reference materials and qualification rate.

Element	ΔlgC		λ		Number of certified reference materials	Number of qualified analysis	Qualification rate
	Max.	Min.	Max.	Min.			
As	–0.004	–0.03	0.022	0.016	13	13	100%
Hg	0.02	–0.002	0.075	0.031	13	13	100%
Cd	0.067	0.055	0.038	0.009	13	13	100%
Pb	0.058	0.025	0.073	0.031	13	13	100%
Cr	–0.009	–0.032	0.067	0.043	13	13	100%
Ni	0.004	–0.014	0.048	0.035	13	13	100%
Cu	–0.007	–0.014	0.036	0.024	13	13	100%
Zn	0.021	0.008	0.022	0.015	13	13	100%

Note: $\Delta lgC = lgC_i - lgC_s$, $\lambda = [\sum(lgC_i - lgC_s)^2]^{1/2} / (n - 1)$, where ΔlgC is the logarithm difference; λ is the degree of precision; Ci is the measured value of certified reference material; Cs is the standard value; n is the number of certified reference materials.

Table S3. Statistics of chemical analysis for repeatedly-analyzed samples and qualification rate.

Element	Relative difference (%)		Number of repeatedly analyzed samples	Number of Qualified analysis	Qualification rate (%)
	Max.	Min.			
As	7.29	–7.87	7	7	100
Hg	13.73	–7.01	7	7	100
Cd	1.42	–39.59	7	7	100
Pb	8.66	–29.09	7	7	100
Cr	5.67	–4.72	7	7	100
Cu	11.16	–11.16	7	7	100
Zn	6.3	–4.9	7	7	100
Pb	8.66	–29.09	7	7	100

Table S4. Statistics of Kolmogorov-Smirnov test for data of elements and PLI.

Variable	Number of samples	Mean	Standard deviation	Kolmogorov-Smirnov Z	Asymptotic significance
As	137	11.65	1.16	1.140	0.149
Hg	137	0.04	0.02	1.326	0.060
Cd	137	0.13	0.05	1.916	0.001
Pb	137	23.16	2.49	1.106	0.173
Cr	137	67.87	4.06	0.758	0.613
Ni	137	29.96	2.51	0.963	0.312
Cu	137	25.02	3.11	1.674	0.007
Zn	137	70.83	13.16	2.618	0.000
PLI	137	1.35	0.27	0.757	0.616
LgCu	137	1.40	0.05	1.339	0.056
LgCd	137	-0.92	0.15	1.648	0.009
LgZn	137	1.85	0.06	1.948	0.001

Table S5. Statistics of semi-variogram fitting results for elements and PLI.

Element	Model	C_0	C_0+C	Range	RSS	R^2	$C_0/(C_0+C)$
As	Linear	1.129018	1.572157	11,305.8934	0.41	0.385	71.81%
	Exponential	0.227	1.441	2790	0.214	0.679	15.75%
	Spherical	0.108	1.418	1840	0.234	0.65	7.62%
	Gaussian	0.496	1.424	1870.6149	0.225	0.663	34.83%
Hg	Linear	0.000295	0.000518	11,305.8934	1.28×10^{-08}	0.833	56.95%
	Exponential	0.000239	0.000518	15210	8.20×10^{-09}	0.893	46.14%
	Spherical	0.000244	0.000489	10200	9.70×10^{-09}	0.887	49.90%
	Gaussian	0.000323	0.000647	19,589.4946	1.88×10^{-08}	0.76	49.92%
Cr	Linear	15.06363	18.502225	11,305.8934	53	0.226	81.42%
	Exponential	0.01	17.5	1800	22.3	0.677	0.06%
	Spherical	1.54	17.44	1640	20.2	0.706	8.83%

	Gaussian	3.38	17.42	1333.6791	20.6	0.7	19.40%
Ni	Linear	4.985013	6.973274	11,305.8934	5.7	0.475	71.49%
	Exponential	0.7	6.291	2190	4.39	0.597	11.13%
	Spherical	0.16	6.231	1580	4.36	0.6	2.57%
	Gaussian	0.81	6.223	1281.7176	4.39	0.596	13.02%
Cu	Linear	0.002502	0.002643	11,305.8934	1.16×10^{-06}	0.022	94.67%
	Exponential	0.000404	0.002628	1380	9.88×10^{-07}	0.176	15.37%
	Spherical	0.000178	0.002596	900	1.08×10^{-06}	0.085	6.86%
	Gaussian	0.000454	0.002598	779.4229	1.08×10^{-06}	0.087	17.47%
PLI	Linear	0.047481	0.080257	11,305.8934	1.85×10^{-03}	0.432	59.16%
	Exponential	0.0112	0.074	5640	6.68×10^{-04}	0.795	15.14%
	Spherical	0.025	0.0738	6150	5.10×10^{-04}	0.843	33.88%
	Gaussian	0.0321	0.0737	5161.5114	5.62×10^{-04}	0.827	43.55%

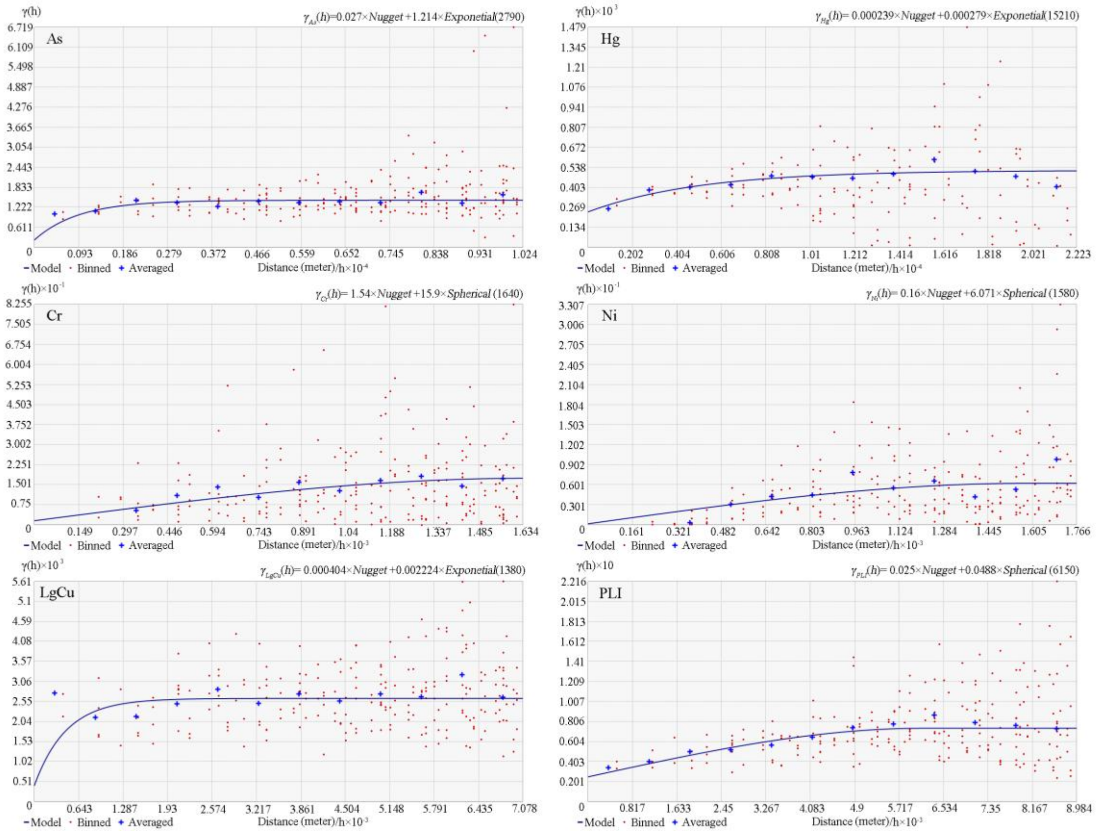

Figure S1. Semi-variograms for elements and PLI interpolation.
